# A new Simple Endoscopic Score for Ulcerative Colitis – the SES-UC

**DOI:** 10.3389/fgstr.2024.1468394

**Published:** 2024-12-13

**Authors:** Viktoria Bergqvist, Peter Gedeon, Erik Hertervig, Jan Marsal

**Affiliations:** ^1^ Department of Gastroenterology, Skane University Hospital, Lund/Malmö, Sweden; ^2^ Section of Medicine, Department of Clinical Sciences, Lund University, Lund, Sweden; ^3^ Immunology Section, Department of Experimental Medical Science, Lund University, Lund, Sweden

**Keywords:** disease activity, disease extent, disease monitoring, endoscopic score, ulcerative colitis

## Abstract

**Background & objectives:**

Endoscopy is the current gold standard for evaluation of disease activity in ulcerative colitis. The Mayo Endoscopic Subscore (MES) is commonly used for quantifying disease activity, but it has several weaknesses. Numerous new endoscopic indices have been developed but none of these have been widely implemented, likely due to limited feasibility. The primary objective of this study was thus to develop a simple, reliable, endoscopic index for ulcerative colitis. Secondary objectives were to evaluate and compare the MES, the Ulcerative Colitis Endoscopic Index of Severity (UCEIS), and the Ulcerative Colitis Colonoscopic Index of Severity (UCCIS), as well as examining the agreement between full colonoscopy and sigmoidoscopy.

**Methods:**

Consecutive adult ulcerative colitis patients had their routine colonoscopies video recorded, each edited into five shorter segment-specific video sequences. In parallel, blood, fecal, and mucosal samples were collected, together with data on symptoms and quality-of-life. The video sequences were scored by three gastroenterologists and one resident gastroenterologist according to a form comprising six endoscopic disease activity descriptors and an overall endoscopic disease severity assessment.

**Results:**

One hundred unique video sequences from twenty patients were each evaluated three times by four assessors, generating a total of 7200 unique segment-specific data-points for the six descriptors and 1200 unique assessments of overall endoscopic disease severity. The intra- and interobserver agreement for the individual descriptors were overall *moderate* to *very good*. The MES, UCEIS, and UCCIS performed similarly with the latter being slightly superior in terms of reliability and correlation to biomarkers of disease activity. The descriptor *vascular pattern* was the best discriminator at the lower end of the disease activity spectrum, whereas the descriptor *ulcers* was the best at the medium to high end. These two descriptors were combined into a new index, the Simple Endoscopic Score for Ulcerative Colitis (SES-UC), which displayed similar levels of reliability and accuracy as the established indices. Finally, comparison of sigmoidoscopy and colonoscopy showed that up to 38% of patients had their most inflamed segment located proximally to the sigmoid colon.

**Conclusions:**

We propose a new simplified endoscopic index for ulcerative colitis, the SES-UC, which is based on the two descriptors *vascular pattern* and *ulcers*. The performance of the SES-UC was similar to, and in some regards better than, that of the established indices (MES, UCEIS, and UCCIS). This together with its simplicity makes SES-UC a candidate index for use in clinical practice as well as in clinical studies.

## Introduction

Ulcerative colitis (UC) is a chronic inflammatory condition of the bowel and is one of the two main types of inflammatory bowel diseases (IBD), the other one being Crohn’s disease (CD). In IBD, endoscopy plays a pivotal role in determining diagnosis, disease monitoring and evaluating treatment effect. Colonoscopy is considered the gold standard for evaluation of inflammatory activity and is frequently used both in clinical trials and routine clinical practice.

The current era of IBD management involves access to numerous and expensive targeted therapies. This, together with an increased understanding of the prognostic value of endoscopic healing, has attributed endoscopic evaluation even greater significance. Other tools for evaluation of inflammatory activity include measurements of biochemical markers, histology, symptoms, and quality-of-life.

The Mayo Endoscopic Subscore (MES) is part of the full Mayo score and is the most widely used endoscopic index for assessment of UC disease activity (1, 2). The MES entails a qualitative assessment of the disease activity, categorized as normal/inactive, mild, moderate, or severe. Since endoscopic evaluation may not be performed by the same endoscopist from time to time, and also, may not be performed by the treating physician, it is important to have a solid assessment tool for communication of endoscopic inflammatory activity. A useful index should be accurate (i.e. the instrument should assess what it is intended to measure), reliable (i.e. not changing upon retesting when no change is expected, including low intra- and interobserver variability), be responsive (i.e. able to detect change in disease activity in an individual over time), be of high resolution (i.e. able to detect subtle yet meaningful differences in disease activity), be simple and easy-to-use in clinical practice as well as feasible for use in clinical trials, and it should be as free as possible of ambiguities when used in interpersonal communication (3–7). Unfortunately, the MES, although widely used, fails to fulfill several of these sought-after criteria (8–10). Numerous initiatives have been taken to develop new endoscopic indices for assessment of inflammatory activity in UC, of which the Ulcerative Colitis Endoscopic Index of Severity (UCEIS), and the Ulcerative Colitis Colonoscopic Index of Severity (UCCIS), are the most comprehensively validated and widely accepted (11–15). A common denominator for most UC endoscopic scores is that they comprise a number of descriptors that are considered to reflect inflammatory activity in different ways (e.g., mucosal erythema, obliterated vascular pattern, granularity, friability, bleeding and erosions/ulcers), and that may be combined and graded in various ways to make up an endoscopic index. The UCEIS comprises vascular pattern (0-2), bleeding (0-3) and erosions/ulcers (0-3), generating a total score of 0-8. The UCCIS consists of four parameters, namely vascular pattern (0-2), granularity (0-2), bleeding/friability (0-2) and ulcers (0-4), each multiplied by a specific weighting factor. An important difference between the MES and UCEIS as compared to the UCCIS is that the MES and UCEIS are designed to evaluate the most inflamed colonic segment at sigmoidoscopy, whereas the UCCIS was designed to evaluate the total inflammatory burden of all colonic segments. However, the UCCIS may be calculated for single segments too, generating a score which we in this study designated segment-specific UCCIS (sUCCIS). Few studies have investigated the potential benefits of a complete colonoscopy as compared to a sigmoidoscopy, and available results on the topic are contradictory (16–18). In recent years several additional indices reflecting the disease burden of the entire colon have been developed in addition to the UCCIS, including the tU (sum of the UCEIS scores for all large bowel segments), the S-MES (sum of the Mayo endoscopic subscores for all large bowel segments), the Modified Mayo Endoscopic Score (MMES), the panMayo score, and the DUBLIN score which are based on the MES, and finally the UCSEI (19–24). The TIGER score is a novel index which combines the MES and the SES-CD scores with an addition of bonus points for more severely inflamed segments; reflects the whole ileocolonic disease burden; and may be used for both UC and CD (1, 25, 26). The Paddington International Virtual Chromoendoscopy Score (PICaSSO) score, developed in 2017, makes use of high-definition virtual electronic chromoendoscopy and has been shown to correlate better to histopathological scores than the MES and UCEIS (27, 28). In recent years, artificial intelligence (AI) has been applied to assist endoscopic evaluation of disease activity in IBD with promising results (29–31). AI-driven tools will most certainly become part of routine clinical evaluation of IBD activity in a not-too-distant future, but right now an improvement of our current tools is warranted (30).

The primary objective of the present study was to develop a simple yet reliable and sensitive endoscopic UC activity index. Secondary objectives included a) to compare the MES/S-MES, the UCEIS/tU, and the sUCCIS/UCCIS in terms of reproducibility, and the relation to histology and inflammatory biomarkers, as well as symptom levels and health-related quality-of-life (HR-QoL), and b) to investigate the potential benefits of a complete colonoscopy as compared to a sigmoidoscopy.

## Methods

We recruited consecutive adult UC patients, diagnosed according to conventional criteria, scheduled for a routine clinical practice colonoscopy at the Skane University Hospital endoscopy unit in Lund, Sweden, to the study. Patients were included irrespective of disease activity level, disease duration, and current therapy. Rectally administered topical treatment was allowed. The study protocol was approved by a Regional Ethics Committee in Sweden (Reference No. 2014-277-32). The study was conducted in accordance with the ethical principles of the Helsinki declaration. Written informed consent was collected from all patients prior to inclusion.

All patients received standard bowel preparation and underwent a complete colonoscopy including intubation of the terminal ileum. Video recording was performed during withdrawal of the instrument. Each video recording was edited into five shorter sequences per patient representing the cecum/ascending, transverse, descending, and sigmoid colon, and the rectum. The recorded video sequences were reviewed in random order by three experienced gastroenterologists and by one resident gastroenterologist at three separate time-points with a two-month interval between each review. After the first read which was not included in the study, a consensus meeting was held in order to harmonize assessments and interpretation of descriptor definitions. Investigators reviewed the recorded sequences independently, and blinded to clinical, laboratory and histological information concerning the patients. Read two and three were used to evaluate intraobserver agreement whereas all other analyses represent the last read. The recorded video sequences were evaluated for the individual descriptors including erythema (scored 0-2), vascular pattern (0-2), granularity (0-2), friability/contact bleeding (0-2), bleeding (0-3), and ulcers (0-3/0-4 as appropriate), as well as for the MES, followed by calculation of the indices S-MES, UCEIS, tU (sum of the UCEIS scores for all five segments), sUCCIS, and UCCIS (**Table 1**) (1, 11, 13, 19, 20). Mucosal contact friability testing was performed at the discretion of the endoscopist performing the colonoscopy using closed biopsy forceps and/or by evaluating the effects of contact between the colonoscope and mucosa.

**Table 1:** Definitions and scoring of items that were assessed for each video sequence.

		Index score	
Item	Grade	UCEIS	UCCIS
Erythema			
Absent	0		
Mild	1		
Marked/pronounced	2		
Vascular pattern			
Normal, or slightly increased	0	0	0
Partial loss	1	1	1
Absent	2	2	2
Granularity			
Absent/normal, smooth surface/ sharp light reflex	0		0
Fine, micronodular surface/scattered light reflex	1		1
Coarse, with pronounced mucosal nodularity	2		2
Friability (contact bleeding)			
Absent	0		0
Intramucosal/diminutive bleeding following light touch	1		1
Evident bleeding following light touch	2		2
Bleeding			
Absent	0	0	
Streaks of coagulated blood/petechiae/intramucosal bleeding	1	1	
Limited amount of free blood in the lumen	2	2	
Oozing blood and/or marked amount of free blood in lumen	3	3	
Ulcers			
Absent	0	0	0
Erosions or pinpoint ulcerations (≤5 mm)	1	1	1
Larger superficial ulcerations (>5 mm)	2	2	2
Deeper excavated ulcers	3	3	3
Diffusely ulcerated mucosa (>30% of surface)	3		4
Mayo Endoscopic Subscore			
Normal mucosa/inactive disease	0		
Mild disease activity	1		
Moderate disease activity	2		
Severe disease activity	3		

UCCIS, Ulcerative Colitis Colonoscopic Index of Severity; UCEIS, Ulcerative Colitis Endoscopic Index of Severity.

Data on patient age, gender, age at disease onset and disease extent according to the Montreal classification, previous and current IBD therapy were recorded. Laboratory tests including plasma C-reactive protein (CRP) and blood hemoglobin (Hb) levels, and fecal calprotectin levels were collected. Mucosal biopsies for histopathological analysis were collected during the colonoscopy. The histological degree of inflammation was categorized as normal, mild, moderate, or severe, and correspondingly scored 0-3 according to the modified Sandborn UC histology index (grade 0 and 1 merged) (32). The aggregate large bowel histological degree of inflammation was calculated by summing the modified Sandborn UC histology index scores for all five large bowel segments. For the latter, missing data imputation (9.4%) was performed using the valid surrounding neighbor values average method (33).

The Simple Clinical Colitis Activity Index (SCCAI) was used for symptom evaluation and the Short Health Scale (SHS) was used to evaluate HR-QoL (34, 35). The SHS consists of four Likert-type items (i.e. severity of symptoms, interference with daily life activities, disease-related worry, and general well-being) that each is scored 0-5, representing a range from no impact to high impact on HR-QoL. A total SHS score was also generated by calculating the sum of the four item scores, with a total score range of 0-20.

### Statistical methods

Statistical analyses were carried out using SPSS statistics for Mac OS X version 29.0.1.1 (IBM Corp.). GraphPad Prism 10.2.0 for Mac OS X (GraphPad Software, Inc.) was used to graph data. Weighted kappa (*κ*) statistics were used to calculate intra- and interobserver agreement. Data are presented as median *κ* value with a 95% confidence interval. Strength of agreement was categorized as poor (*κ* 0.00-0.20), fair (*κ* 0.21–0.40), moderate (*κ* 0.41–0.60), good (*κ* 0.61–0.80), or very good (*κ* 0.81-1.00) (36). Correlation between single descriptors and indices, respectively, with various indicators of disease activity including biochemical markers of inflammatory activity, histopathology, the SCCAI and the SHS, respectively, were carried out using the Spearman’s rank-order or Pearson’s correlation analysis, depending on data characteristics and distribution, per each separate large bowel segment or per all five large bowel segments collectively as indicated. A p-value <0.05 was considered statistically significant. To investigate whether a full colonoscopy provided additional information as compared to a sigmoidoscopy, we calculated the proportion of cases where the most inflamed segment was located proximally to the sigmoid colon, and thus not visualized at sigmoidoscopy.

## Results

### Patient characteristics and location of inflammatory maximum

Twenty patients with ulcerative colitis were included in the study. Detailed data on patient characteristics including age at study inclusion and diagnosis, respectively, gender, disease extent, and IBD therapy are summarized in **Table 2**. At colonoscopy, four patients (20%) displayed proctitis, three patients (15%) had left-sided colitis and thirteen patients (65%) presented with extensive colitis. The mean age of the patients at study inclusion was 41.5 years (range 18-76); 8 were males and 12 were females. Six patients were treated with systemic corticosteroids at the time of the examination and one patient was treated with anti-TNF therapy. Two patients were treated with topical treatment with 5-ASA and topical corticosteroids, respectively.

**Table 2:** Demographics, disease characteristics, and therapy data on study subjects.

Demographics	
Age at inclusion, Mean (range)	41.5 (18-76)
Age at diagnosis, Mean (range)	38.7 (18-76)
Gender male:female (%)	40:60

Montreal classification, n (%)	
*Age at diagnosis*	
A1 (<16 years)	0 (0%)
A2 (17-40 years)	12 (60%)
A3 (>40 years)	8 (40%)
Disease extension	
E1 (proctitis)	4 (20%)
E2 (left-sided)	3 (15%)
E3 (extensive)	13 (65%)

Prior IBD therapy, n (%)	
Mesalazine	16 (80%)
Sulfasalazine	1 (5%)
Thiopurines*	5 (25%)
Anti-TNFα agent	2 (10%)
Systemic corticosteroids	12 (60%)
Budesonide	1 (5%)
Topical treatment	
Mesalazine	11 (55%)
Corticosteroids	5 (25%)

Current IBD therapy, n (%)	
Mesalazine	14 (70%)
Thiopurines*	3 (15%)
Anti-TNFα agent	1 (5%)
Systemic corticosteroids	6 (30%)
Budesonide	1 (5%)
Topical treatment	
Mesalazine	1 (5%)
Corticosteroids	1 (5%)

IBD, inflammatory bowel disease; n, number of patients; TNFα, tumor necrosis factor α.

*Includes azathioprine and 6-mercaptopurine.

Interestingly, 25%, 35%, and 38% of patients with left-sided or extensive colitis had their most inflamed segment located proximally from the sigmoid colon when inflammatory activity was quantified by the MES, UCEIS, and sUCCIS, respectively.

### Intraobserver and interobserver agreement analyses of endoscopic disease activity descriptors and endoscopic indices

We analyzed intra- and interobserver agreement for the various endoscopic disease activity descriptors and endoscopic indices using kappa (*κ*) statistics. For the endoscopic indices, analyses were performed both for each colonic segment separately and for the whole large bowel. All assessed descriptors demonstrated *good* to *very good* intraobserver agreement in all segments, except for *erythema* and *ulcers* for which agreement in the sigmoid colon was just below the cut-off value of 0.60 reflecting *good* agreement (*κ* 0.59 and 0.57, respectively) (**Table 3**). However, comparing the mean intraobserver *κ* values across all five segments, *erythema* and *ulcers* showed similar values as *friability* and *bleeding*, whereas *vascular pattern* and *granularity* showed higher values than the other descriptors (**Table 3**). When examining segment-specific intraobserver agreement for endoscopic indices the sUCCIS displayed the highest *κ* values and MES the lowest, with UCEIS in between the two (**Table 3**). Intraobserver agreement was *good* for the pancolonic S-MES (*κ* 0.71) and tU (*κ* 0.78), whereas for the UCCIS agreement was *very good* (*κ* 0.87) (**Table 3**).

**Table 3:** Segment-specific and pancolonic intraobserver agreement (kappa values) for individual descriptors and composite endoscopic scores.

Parameter [*κ* (95% CI)]	Cecum/Asc.	Transverse	Descending	Sigmoid	Rectum	Mean *κ* (SD)	Pancolonic κ
Erythema	0.74 (0.51-0.97)	0.71 (0.46-0.95)	0.74 (0.53-0.94)	0.59 (0.31-0.86)	0.72 (0.47-0.98)	0.70 (0.06)	–
Vascular pattern	0.82 (0.61-1.00)	0.78 (0.57-1.00)	0.92 (0.79-1.00)	0.72 (0.49-0.97)	0.87 (0.68-1.00)	0.82 (0.08)	–
Granularity	0.82 (0.59-1.00)	0.85 (0.67-1.00)	0.86 (0.70-1.00)	0.84 (0.66-1.00)	0.74 (0.52-0.95)	0.82 (0.05)	–
Friability	0.79 (0.57-1.00)	0.72 (0.48-0.95)	0.68 (0.43-0.92)	0.61 (0.39-0.84)	0.82 (0.60-1.00)	0.72 (0.08)	–
Bleeding	0.74 (0.51-0.98)	0.80 (0.56-1.00)	0.69 (0.48-0.90)	0.69 (0.45-0.94)	0.63 (0.37-0.88)	0.71 (0.06)	–
Ulcers	0.80 (0.56-1.00)	0.74 (0.52-0.95)	0.72 (0.50-0.93)	0.57 (0.33-0.82)	0.72 (0.50-0.90)	0.71 (0.08)	–
MES*/S-MES^#^	0.72 (0.48-0.96)	0.73 (0.53-0.92)	0.78 (0.59-0.96)	0.62 (0.37-0.88)	0.82 (0.67-0.98)	0.73 (0.08)	0.71 (0.59-0.82)
UCEIS*/tU^#^	0.83 (0.68-0.96)	0.78 (0.65-0.90)	0.80 (0.67-0.92)	0.66 (0.48-0.83)	0.74 (0.61-0.87)	0.76 (0.07)	0.78 (0.69-0.87)
sUCCIS/*UCCIS^#^	0.82 (0.67-0.97)	0.84 (0.73-0.96)	0.81 (0.69-0.91)	0.69 (0.55-0.83)	0.78 (0.67-0.89)	0.79 (0.06)	0.87 (0.77-0.97)

Asc, ascending; CI, confidence interval; *κ*, kappa value of agreement; MES, Mayo Endoscopic Subscore; SD, standard deviation; sUCCIS, segment-specific UCCIS; S-MES, sum of Mayo Endoscopic Subscores; tU, total UCEIS; UCCIS, Ulcerative Colitis Colonoscopic Index of Severity; UCEIS, Ulcerative Colitis Endoscopic Index of Severity.

*Segment-specific; ^#^Pancolonic.

Interobserver *κ* values were overall somewhat lower than intraobserver *κ* values, but still corresponding to mainly *moderate* (*κ* 0.41–0.60) or *good* (*κ* 0.61–0.80) agreement. Interobserver agreement for individual descriptors and composite indices showed a tendency toward higher agreement in proximal colonic segments and lower agreement in more distal segments (**Table 4**). Comparing the mean interobserver *κ* values across all five segments, the descriptors *erythema, granularity, friability*, *bleeding*, and *ulcers* showed similar values, whereas *vascular pattern* showed a higher degree of agreement than the others (**Table 4**). As for the segment-specific interobserver agreement for endoscopic indices the sUCCIS and UCEIS showed similar *κ* values and MES somewhat lower values (**Table 4**). Interobserver agreement for the pancolonic UCCIS was *good* (*κ* 0.64), while it was *moderate* for the S-MES (*κ* 0.60) and tU (*κ* 0.58) (**Table 4**).

**Table 4:** Segment-specific and pancolonic interobserver agreement (kappa values) for individual descriptors and composite endoscopic scores.

Parameter [*κ* (95% CI)]	Cecum/Asc.	Transverse	Descending	Sigmoid	Rectum	Mean *κ* (SD)	Pancolonic *κ*
Erythema	0.62 (0.33-0.91)	0.67 (0.41-0.93)	0.72 (0.50-0.94)	0.38 (0.08-0.71)	0.55 (0.24-0.85)	0.59 (0.13)	–
Vascular pattern	0.58 (0.32-0.84)	0.64 (0.40-0.87)	0.78 (0.60-0.96)	0.63 (0.37-0.87)	0.66 (0.47-0.86)	0.66 (0.07)	–
Granularity	0.62 (0.34-0.90)	0.62 (0.38-0.86)	0.69 (0.47-0.90)	0.46 (0.21-0.72)	0.39 (0.13-0.65)	0.56 (0.13)	–
Friability	0.63 (0.34-0.90)	0.66 (0.43-0.90)	0.58 (0.35-0.81)	0.47 (0.25-0.70)	0.51 (0.26-0.75)	0.57 (0.08)	–
Bleeding	0.64 (0.40-0.86)	0.54 (0.30-0.78)	0.48 (0.23-0.75)	0.49 (0.21-0.80)	0.52 (0.23-0.79)	0.53 (0.06)	–
Ulcers	0.64 (0.43-0.84)	0.57 (0.31-0.83)	0.50 (0.21-0.78)	0.39 (0.18-0.60)	0.56 (0.32-0.80)	0.53 (0.09)	–
MES*/S-MES^#^	0.64 (0.37-0.90)	0.66 (0.45-0.87)	0.56 (0.35-0.77)	0.44 (0.24-0.66)	0.59 (0.41-0.81)	0.58 (0.09)	0.60 (0.48-0.75)
UCEIS*/tU^#^	0.68 (0.44-0.87)	0.64 (0.46-0.78)	0.64 (0.46-0.80)	0.51 (0.34-0.68)	0.62 (0.48-0.75)	0.62 (0.06)	0.58 (0.47-0.72)
sUCCIS*/UCCIS^#^	0.67 (0.49-0.86)	0.69 (0.56-0.84)	0.63 (0.48-0.77)	0.56 (0.41-0.70)	0.61 (0.51-0.72)	0.63 (0.05)	0.64 (0.51-0.77)

Asc, ascending; CI, confidence interval; *κ*, kappa value of agreement; MES, Mayo Endoscopic Subscore; SD, standard deviation; sUCCIS, segment-specific UCCIS; S-MES, sum of Mayo Endoscopic Subscores; tU, total UCEIS; UCCIS, Ulcerative Colitis Colonoscopic Index of Severity; UCEIS, Ulcerative Colitis Endoscopic Index of Severity.

*Segment-specific; ^#^Pancolonic.

### Correlations between endoscopic indices and biochemical markers of inflammatory activity, histological degree of inflammation, symptom levels, and health-related quality-of-life

To examine how the individual endoscopic descriptors and the various endoscopic scores reflected disease activity, we correlated them with a number of measurement tools that are used to quantitate disease activity. These included histology and biomarkers (plasma CRP, blood Hb, and fecal calprotectin) which are segment-specific and nonsegment-specific, respectively, objective measurements, and symptom levels and health-related quality-of-life which are subjective measurements. The six descriptors examined, and the MES and the UCEIS that are segment-specific by their original design, and the sUCCIS, all displayed statistically significant and strong or very strong correlations with the histological degree of inflammation in all colonic segments (**Table 5**). Furthermore, the previously established endoscopic scores (i.e., MES, UCEIS, and UCCIS) all correlated significantly and strongly or very strongly with the levels of plasma CRP and fecal calprotectin (**Table 6**). In contrast, blood Hb and symptom levels assessed using the SCCAI correlated poorly to the endoscopic descriptors and scores (**Table 6**).

**Table 5:** Segment-specific correlations between individual endoscopic disease activity descriptors or endoscopic scores, and the histological degree of inflammation.

Histological inflammation	Cecum/Asc.		Transverse		Descending		Sigmoid		Rectum	
	*r*_s_	*p*	*r*_s_	*p*	*r*_s_	*p*	*r*_s_	*p*	*r*_s_	*p*
Disease activity descriptor										
Erythema	0.830	<0.001	0.752	0.001	0.872	<0.001	0.864	<0.001	0.672	0.003
Vascular pattern	0.833	<0.001	0.719	0.002	0.894	<0.001	0.889	<0.001	0.614	0.009
Granularity	0.917	<0.001	0.840	<0.001	0.836	<0.001	0.869	<0.001	0.683	0.002
Friability	0.881	<0.001	0.788	<0.001	0.721	0.002	0.678	0.008	0.482	0.050
Bleeding	0.765	0.001	0.788	<0.001	0.840	<0.001	0.779	0.001	0.617	0.008
Ulcers	0.725	0.002	0.670	0.005	0.742	0.002	0.802	0.001	0.717	0.001
Endoscopic score										
MES*	0.855	<0.001	0.778	<0.001	0.835	<0.001	0.795	0.001	0.630	0.007
UCEIS*	0.827	<0.001	0.794	<0.001	0.863	<0.001	0.882	<0.001	0.653	0.004
sUCCIS*	0.831	<0.001	0.785	<0.001	0.817	<0.001	0.899	<0.001	0.732	0.001

Asc, ascending; MES, Mayo Endoscopic Subscore; *p*, p-value; *r*_s_, Spearman’s rank correlation coefficient; sUCCIS, segment-specific UCCIS; UCCIS, Ulcerative Colitis Colonoscopic Index of Severity; UCEIS, Ulcerative Colitis Endoscopic Index of Severity.

*Segment-specific.

**Table 6:** Segment-specific and pancolonic correlation analyses between endoscopic scores and disease activity levels measured by inflammatory biomarkers or symptom scores.

Parameter	Cecum/Asc.		Transverse		Descending		Sigmoid		Rectum		Pancolonic	
	*r*_s_	*p*	*r*_s_	*p*	*r*_s_	*p*	*r*_s_	*p*	*r*_s_	*p*	*r*_s_	*p*
Plasma C-reactive protein												
S-MES^#^	–	–	–	–	–	–	–	–	–	–	0.816	<0.001
tU^#^	–	–	–	–	–	–	–	–	–	–	0.786	<0.001
UCCIS^#^	–	–	–	–	–	–	–	–	–	–	0.810	<0.001
Blood hemoglobin												
S-MES^#^	–	–	–	–	–	–	–	–	–	–	-0.469	0.079
tU^#^	–	–	–	–	–	–	–	–	–	–	-0.423	0.117
UCCIS^#^	–	–	–	–	–	–	–	–	–	–	-0.392	0.166
Fecal calprotectin												
S-MES^#^	–	–	–	–	–	–	–	–	–	–	0.625	0.019
tU^#^	–	–	–	–	–	–	–	–	–	–	0.712	0.006
UCCIS^#^	–	–	–	–	–	–	–	–	–	–	0.864	<0.001
Symptom score (SCCAI)												
MES*/S-MES^#^	0.017	0.959	0.188	0.579	0.380	0.248	0.475	0.140	0.520	0.101	0.405	0.217
UCEIS*/tU^#^	-0.032	0.925	0.199	0.558	0.169	0.620	0.439	0.177	0.579	0.062	0.347	0.295
sUCCIS*/UCCIS^#^	0.061	0.859	0.220	0.515	0.438	0.178	0.597	0.052	0.619	0.042	0.516	0.104

Asc, ascending; MES, Mayo Endoscopic Subscore; *p*, p-value; *r*_s_, Spearman’s rank correlation coefficient; SCCAI, Simple Clinical Colitis Activity Index; sUCCIS, segment-specific UCCIS; S-MES, sum of Mayo Endoscopic Subscores; tU, total UCEIS; UCCIS, Ulcerative Colitis Colonoscopic Index of Severity; UCEIS, Ulcerative Colitis Endoscopic Index of Severity.

*Segment-specific; #Pancolonic.

Similarly, the correlations between HR-QoL as assessed by the SHS and the endoscopic scores, including the segment-specific MES, UCEIS, and sUCCIS and the pancolonic S-MES and tU were poor (**Table 7**). The pancolonic UCCIS however differed from this pattern and showed significant correlations with HR-QoL levels which was true for both the separate SHS items and the total SHS score (**Table 7**). Interestingly, the overall correlation between endoscopic scores and SHS levels was better for all indices in the sigmoid colon as compared to other colonic segments (**Table 7**). Finally, the SHS items *worry* and *general well-being* correlated generally somewhat better with the endoscopic indices as compared to the SHS items *symptoms* and *activity* (**Table 7**).

**Table 7:** Segment-specific and pancolonic correlations between endoscopic scores and health-related quality-of-life as defined by the SHS.

Parameter	Cecum/Asc.		Transverse		Descending		Sigmoid		Rectum		Pancolonic		
	*r*_s_	*p*	*r*_s_	*p*	*r*_s_	*p*	*r*_s_	*p*	*r*_s_	*p*	*r*_s_	*p*
SHS HR-QoL symptoms												
MES*/S-MES^#^	-0.059	0.856	0.222	0.488	0.462	0.131	0.542	0.069	0.428	0.165	0.433	0.160
UCEIS*/tU^#^	-0.109	0.735	0.258	0.418	0.283	0.373	0.531	0.076	0.489	0.107	0.426	0.167
sUCCIS*/UCCIS^#^	0.047	0.884	0.342	0.276	0.444	0.149	0.687	0.014	0.547	0.065	0.580	0.048
SHS HR-QoL activity												
MES*/S-MES^#^	0.168	0.602	0.225	0.481	0.548	0.065	0.496	0.101	0.370	0.237	0.458	0.134
UCEIS*/tU^#^	0.117	0.717	0.233	0.466	0.364	0.245	0.527	0.078	0.354	0.259	0.448	0.144
sUCCIS*/UCCIS^#^	0.229	0.474	0.382	0.221	0.500	0.098	0.637	0.026	0.449	0.143	0.594	0.042
SHS HR-QoL worry												
MES*/S-MES^#^	0.246	0.441	0.494	0.103	0.662	0.019	0.740	0.006	0.443	0.149	0.554	0.062
UCEIS*/tU^#^	0.215	0.502	0.541	0.070	0.541	0.070	0.739	0.006	0.414	0.181	0.583	0.047
sUCCIS*/UCCIS^#^	0.291	0.360	0.581	0.047	0.683	0.014	0.778	0.003	0.446	0.147	0.762	0.004
SHS HR-QoL general well-being												
MES*/S-MES^#^	0.346	0.270	0.305	0.336	0.629	0.028	0.759	0.004	0.401	0.196	0.542	0.069
UCEIS*/tU^#^	0.339	0.282	0.308	0.330	0.510	0.090	0.616	0.033	0.411	0.184	0.495	0.102
sUCCIS*/UCCIS^#^	0.349	0.266	0.448	0.144	0.688	0.013	0.730	0.007	0.466	0.127	0.718	0.009
SHS HR-QoL total score												
MES*/S-MES^#^	0.164	0.610	0.286	0.367	0.601	0.039	0.687	0.014	0.447	0.146	0.502	0.096
UCEIS*/tU^#^	0.133	0.681	0.307	0.331	0.435	0.158	0.645	0.023	0.464	0.129	0.508	0.092
sUCCIS*/UCCIS^#^	0.222	0.489	0.421	0.173	0.627	0.029	0.767	0.004	0.533	0.074	0.706	0.010

Asc, ascending; HR-QoL, health-related quality-of-life; MES, Mayo Endoscopic Subscore; *p*, p-value; *r*_s_, Spearman’s rank correlation coefficient; SHS, Short Health Scale; sUCCIS, segment-specific UCCIS; S-MES, sum of Mayo Endoscopic Subscores; tU, total UCEIS; UCCIS, Ulcerative Colitis Colonoscopic Index of Severity; UCEIS, Ulcerative Colitis Endoscopic Index of Severity.

*Segment-specific. #Pancolonic.

### Discriminative capacity of individual descriptors along the inflammatory spectrum

**Figure 1:**
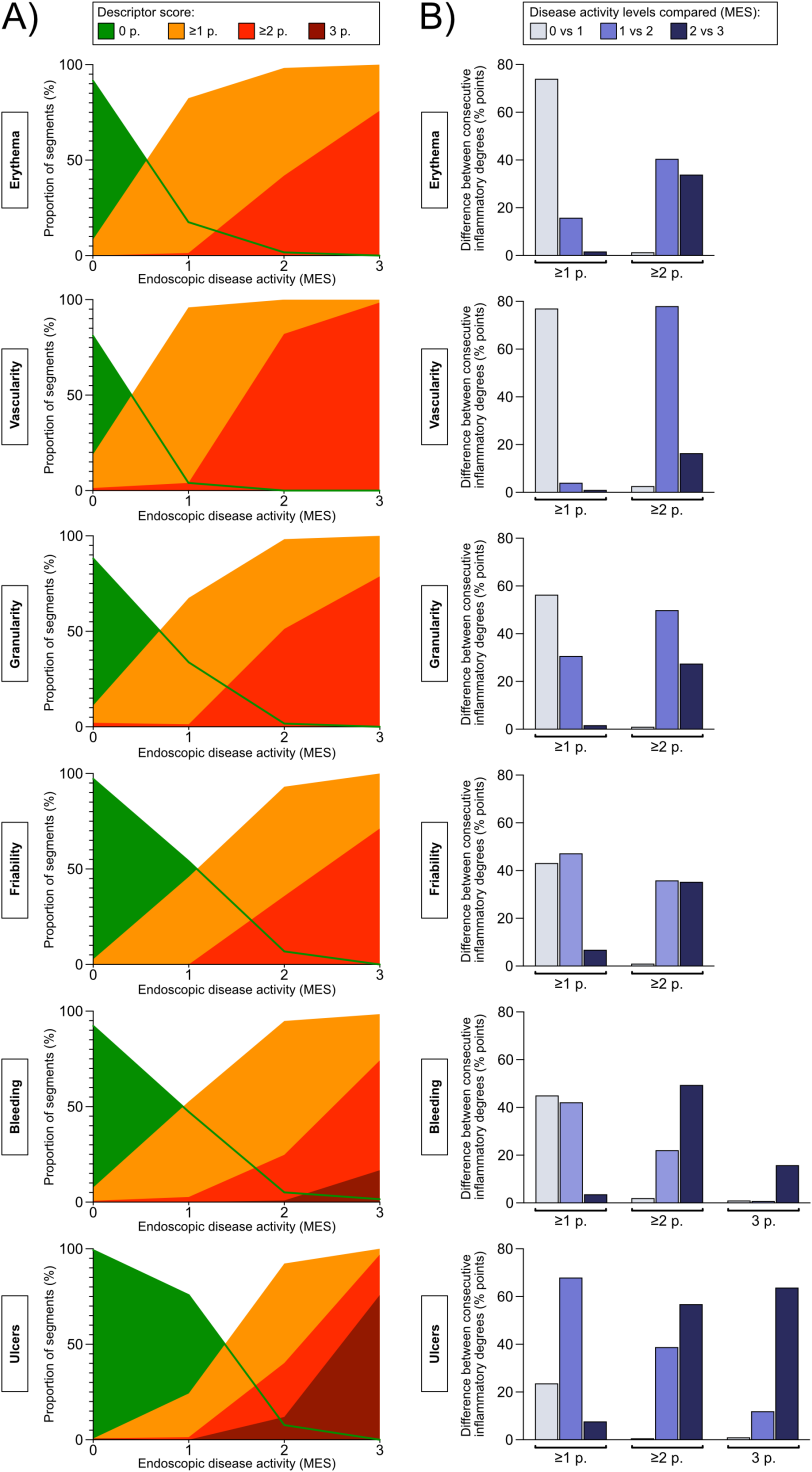
**(A)** Concordance percentages between the Mayo Endoscopic Subscore (levels 0-3, presented on the X-axis) and the endoscopic disease activity descriptors (percentages, presented on the Y-axis). The purpose of the analysis was to examine the discriminatory ability of the various descriptors in mild, moderate, and severe inflammatory activity, respectively. The Mayo Endoscopic Subscore (MES) was used as reference for disease activity. For each MES level (X-axis), the percentage of segments that was given a descriptor-score of 0, ≥1, ≥2, or 3 if applicable, is presented (Y-axis). The descriptor-scores are color-coded according to legend. A) erythema, 0-2 points; B) vascular pattern, 0-2 points; C) granularity, 0-2 points; D) friability, 0-2 points; E) bleeding, 0-3 points; and F) vascularity, 0-3 points. **(B)** The graphs show the difference in the proportion of segments that score ≥1, ≥2, or if applicable 3 points, for the given endoscopic disease activity descriptor between consecutive Mayo Endoscopic Subscore levels (*i.e.* Mayo Endoscopic Subscore 0 versus 1 point, 1 versus 2 points, and 2 versus 3 points). The analysis was performed to examine to what degree a given endoscopic disease activity descriptor can differentiate between two consecutive grades of inflammation as defined by the Mayo Endoscopic Subscore.

**Figure 2:**
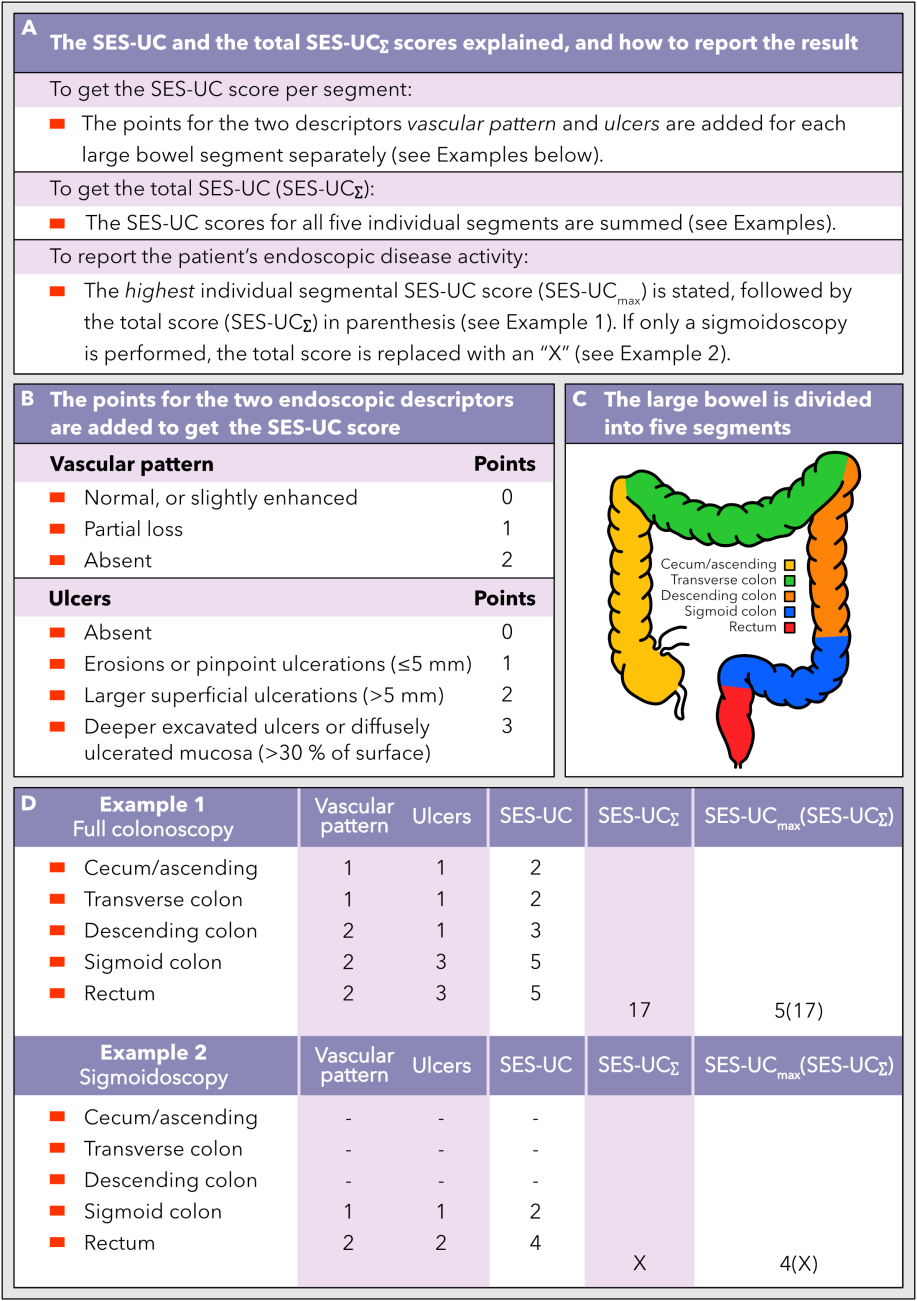
How to calculate and use the new SES-UC score. **(A)** A description of the segmental SES-UC score, the total SES-UC (SES-UC_Σ_), and the reporting format of SES-UC. **(B)** The endoscopic disease activity descriptors that the SES-UC is based on, and how to calculate the SES-UC score. **(C)** The large bowel is divided into five segments when assessing the endoscopic disease activity according to the SES-UC. **(D)** Examples of how the SES-UC and the SES-UC_Σ_ scores are calculated, and how to report the scores, when a full colonoscopy and a sigmoidoscopy, respectively, have been performed.

Given that specific descriptors perform better or worse when it comes to discriminating between more subtle differences in disease activity, in different ranges along the full spectrum of disease severity, the selection of descriptors that comprise a given index affects at what resolution the index will be able to recognize differences or changes in disease activity in the low, medium, and high end of the disease severity spectrum. To examine which descriptors are good at discriminating more subtle differences in the degree of inflammation in mild, moderate, and severe disease activity, respectively, separate descriptors were plotted against MES as reference (**Figure 1A**). Next, we calculated the difference in proportion of single segments that scored ≥1, ≥2, and 3, respectively, according to the specific descriptors, when comparing segments categorized as MES 0 and 1, MES 1 and 2, and MES 2 and 3 (**Figure 1B**). This analysis was performed to elucidate which descriptors reacted the most (i.e. were the most sensitive) when comparing neighboring degrees of inflammatory activity along the disease severity spectrum, and conversely, which descriptors that were insensitive to a shift between two neighboring inflammatory levels. The results indicate that the descriptor *vascular pattern* is the best discriminator at the lower end of the disease severity spectrum, whereas the descriptor *ulcers* is the best at discriminating in the medium and high range of the inflammatory activity spectrum. In addition, the descriptor *vascular pattern* displayed the highest average intra- and interobserver agreement numbers for the five large bowel segments taken together, whereas the descriptor *ulcers* showed similar agreement data as the other descriptors investigated (**Tables 3**, **4**). Based on these results, these two descriptors (*vascular pattern* and *ulcers*) were selected to make up a new index tentatively named the Simple Endoscopic Score for Ulcerative Colitis, SES-UC (**Table 1**; **Figure 2A**).

### Performance of the new proposed Simple Endoscopic Score for Ulcerative Colitis (SES-UC)

We compared the performance of the new simplified index SES-UC to that of the previously established indices including MES, UCEIS, and UCCIS, respectively. The results showed that SES-UC/SES-UC_Σ_ performs in parity with the UCEIS/tU and sUCCIS/UCCIS, and somewhat better than MES/S-MES, in terms of intraobserver agreement, except for the pancolonic score where the UCCIS performed slightly better (**Tables 3**, **8A**). Interobserver agreement values for SES-UC/SES-UC_Σ_ were also similar to those of the other indices (**Tables 4**, **8A**).

**Table 8:** (A) Segment-specific and pancolonic intra- and interobserver agreement (kappa values) for the new simplified endoscopic score SES-UC. (B) Segment-specific and pancolonic correlations between the new simplified endoscopic score SES-UC and the histological degree of inflammation, biochemical markers, symptom levels (SCCAI), and health-related quality-of-life (SHS). (C) Correlations between various measures of disease activity (including the histological degree of inflammation, biochemical markers, symptom levels [SCCAI], and health-related quality-of-life [SHS]) and the SES-UC_max_ or the SES-UC_max_ x SES-UC_∑_.

A.							
SES-UC [*κ* (95% CI)]	Cecum/Asc.	Transverse	Descending	Sigmoid	Rectum	Mean *κ* (SD)	Pancolonic^†^ *κ*
Intraobserver agreement	0.83 (0.64-1.00)	0.76 (0.60-0.93)	0.84 (0.70-0.98)	0.68 (0.51-0.85)	0.78 (0.64-0.93)	0.78 (0.06)	0.77 (0.68-0.88)
Interobserver agreement	0.67 (0.40-0.88)	0.63 (0.45-0.80)	0.66 (0.50-0.83)	0.48 (0.30-0.68)	0.61 (0.46-0.77)	0.61 (0.08)	0.58 (0.44-0.72)

B.												
Correlation analyses (SES-UC)	Cecum/Asc.		Transverse		Descending		Sigmoid		Rectum		Pancolonic^†^	
	*r*_s_	*p*	*r*_s_	*p*	*r*_s_	*p*	*r*_s_	*p*	*r*_s_	*p*	*r*_s_ or *r*_p_^§^	*p*
Histological inflammation	0.762	0.001	0.758	0.001	0.841	<0.001	0.871	<0.001	0.704	0.002	0.828	<0.0001
Plasma C-reactive protein	–	–	–	–	–	–	–	–	–	–	0.837	<0.001
Blood hemoglobin	–	–	–	–	–	–	–	–	–	–	-0.386	0.173
Fecal calprotectin	–	–	–	–	–	–	–	–	–	–	0.818	<0.001
Symptom score (SCCAI)	0.148	0.663	0.188	0.580	0.169	0.620	0.547	0.081	0.589	0.056	0.510	0.109
SHS HR-QoL symptoms	0.047	0.884	0.172	0.593	0.314	0.320	0.674	0.016	0.603	0.038	0.539	0.070
SHS HR-QoL activity	0.287	0.366	0.179	0.578	0.392	0.208	0.617	0.032	0.490	0.106	0.543	0.068
SHS HR-QoL worry	0.294	0.353	0.460	0.133	0.575	0.050	0.773	0.003	0.508	0.092	0.697	0.012
SHS HR-QoL general well-being	0.460	0.132	0.329	0.297	0.548	0.065	0.726	0.007	0.449	0.143	0.733	0.007
SHS HR-QoL total score	0.287	0.366	0.263	0.409	0.473	0.121	0.755	0.005	0.554	0.062	0.676	0.016

C.				
Correlation analyses (SES-UC)	SES-UC_max_		[SES-UC_max_ x SES-UC_Σ_]	
	*r*_s_	*p*	*r*_s_ or *r*_p_^§^	*p*
Histological inflammation (maximal)	0.82	<0.0001	0.77	<0.001
Histological inflammation (aggregate)	–	–	0.81	<0.0001
Plasma C-reactive protein	0.69	<0.001	0.78	<0.001
Blood hemoglobin	-0.26	0.346	-0.45	0.091
Fecal calprotectin	0.62	0.021	0.80	<0.001
Symptom score (SCCAI)	0.42	0.196	0.42	0.193
SHS HR-QoL symptoms	0.50	0.098	0.56	0.061
SHS HR-QoL activity	0.42	0.178	0.50	0.100
SHS HR-QoL worry	0.63	0.033	0.75	0.006
SHS HR-QoL general well-being	0.46	0.135	0.63	0.031
SHS HR-QoL total score	0.52	0.085	0.65	0.026

Asc, ascending; CI, confidence interval; HR-QoL, health-related quality-of-life; *κ*, kappa value of agreement; max, maximal; *p*, p-value; *r*_p_, Pearson’s correlation coefficient; *r*_s_, Spearman’s rank correlation coefficient; SCCAI, Simple Clinical Colitis Activity Index; SD, standard deviation; SES-UC, Simple Endoscopic Score for Ulcerative Colitis; SES-UC_Σ_, total SES-UC; SHS, Short Health Scale.

^†^SES-UC_Σ_; ^§^depending on data characteristics and distribution.

Furthermore, there was a high degree of statistically significant correlation of the SES-UC and SES-UC_Σ_, respectively, to the degree of histological disease activity (**Table 8B**). The same was true for the correlation of SES-UC_Σ_ to plasma CRP and fecal calprotectin (**Table 8B**). In contrast, the correlation of SES-UC_Σ_ to symptom levels (SCCAI) and blood Hb was poor (**Table 8B**). The degree of correlation with plasma CRP, fecal calprotectin, blood Hb, histological disease activity, and symptom-levels (SCCAI) was thus similar for SES-UC_Σ_ as for the other indices (**Tables 5**, **6**, **8B**), except regarding fecal calprotectin where SES-UC_Σ_ and UCCIS showed superior correlation as compared with S-MES and tU.

Finally, we analyzed the correlation between endoscopic scores and HR-QoL as measured by the four SHS items and the total SHS score, respectively (**Tables 7**, **8B**). Among the endoscopic indices, the SES-UC_Σ_ and UCCIS showed the best correlation with the total SHS (HR-QoL) score, as well as with the single SHS items worry and general well-being (**Tables 7**, **8B**). Interestingly, there was a trend toward higher correlation values (*r*) and lower p-values for segments located distally to the splenic flexure (**Tables 7**, **8B**).

Numerous attempts have been made at constructing an endoscopic index that captures both the degree of inflammation and the extent of inflammation in one single number. However, we believe that a single number is not sufficient in clinical practice to describe and communicate the various relevant aspects of the patient’s endoscopic inflammatory burden in ulcerative colitis. Instead, we suggest that two numbers should be used, one that denotes the highest degree of inflammation found at colonoscopy and one that denotes the aggregate degree of inflammation for the whole large bowel. We therefore examined the relationship between the highest SES-UC score (SES-UC_max_) with the highest degree of histological inflammation. The results showed a high degree of statistically significant correlation between the SES-UC_max_ score and the maximal histological degree of inflammation (**Table 8C**). Next, we examined the relationship between the SES-UC_max_ score with plasma CRP and fecal calprotectin, which also showed statistically significant correlation values (**Table 8C**). In contrast, correlations between the SES-UC_max_ score and blood Hb, symptom-levels (SCCAI), and HR-QoL (SHS) were poor (**Table 8C**). Finally, we analyzed the correlation between the SES-UC_max_ score multiplied by SES-UC_Σ_ score ([SES-UC_max_ X SES-UC_Σ_]; see below for rationale) and the maximal histological inflammation, the aggregate histological inflammation, plasma CRP, and fecal calprotectin, respectively. The results showed high and statistically significant correlation values (**Table 8C**). In contrast, correlations between the [SES-UC_max_ X SES-UC_Σ_] score and blood Hb, symptom-levels (SCCAI), and HR-QoL (SHS) were poor or moderate (**Table 8C**).

### The new Simple Endoscopic Score for Ulcerative Colitis (SES-UC)

We thus propose a new simplified endoscopic index for ulcerative colitis, herein named the SES-UC, which is the sum of the points for the two descriptors *vascular pattern* and *ulcers*, and is scored for each large bowel segment separately (**Figures 2A, B**). The total SES-UC (SES-UC_Σ_) is the sum of the SES-UC scores for all five large bowel segments (**Figures 2A, C**). To communicate or record a UC-patient’s endoscopic degree of disease activity as defined by the SES-UC, the highest segmental SES-UC score observed is noted, followed by the SES-UC_Σ_ score in parenthesis (**Figures 2A, D**). If only a sigmoidoscopy has been performed as opposed to a full colonoscopy, the SES-UC_Σ_ score should be replaced with an “X” (**Figures 2A, D**). For calculation examples, see **Figure 2D**.

### Comparison of performance between the SES-UC and the established indices

In order to evaluate the performance of the indices as objectively as possible, we collected the results of the performance indicator analyses, and scored each parameter 0-3 points according to the rank position among the four indices investigated. The index that displayed the highest coefficient number scored 3 and the one with the lowest number scored 0, for each respective parameter. If equal, the score was shared. The points for each index were summed to generate a total performance score (**Table 9**). The results showed that the MES received 2.5 points, the UCEIS 6.5 points, the UCCIS 16.0 points, and the SES-UC 11.0 points. According to this, the UCCIS holds the best overall performance qualities, followed by the SES-UC and the UCEIS, with the MES showing the lowest overall level of performance (**Table 9**).

**Table 9:** Comparison of performance between the SES-UC and the established indices (MES, UCEIS, and UCCIS).

	MES	UCEIS	UCCIS	SES-UC
Intraobserver agreement (*κ*)	0.71	0.78	0.87	0.77
Interobserver agreement (*κ*)	0.60	0.58	0.64	0.58
Histological inflammation (*r*)	0.79	0.83	0.84	0.83
Plasma C-reactive protein (*r*)	0.82	0.79	0.81	0.84
Blood hemoglobin (*r*)	ns	ns	ns	ns
Fecal calprotectin (*r*)	0.63	0.71	0.86	0.82
Symptom Score (SCCAI) (*r*)	ns	ns	ns	ns
SHS HR-QoL total score (*r*)	ns	ns	0.71	0.68
Performance score*	**2.5**	**6.5**	**16.0**	**11.0**

*According to the rank position within the group of the four indices, each performance indicator was scored 0-3 points. The index displaying the highest coefficient number scored 3 and the one with the lowest number scored 0. If equal, the score was shared. The sum of points, i.e. the performance score, is presented in bold for each endoscopic index. HR-QoL, health-related quality-of-life; k, kappa value of agreement; MES, Mayo Endoscopic Subscore; r, correlation coefficient; SCCAI, Simple Clinical Colitis Activity Index; SES-UC, Simple Endoscopic Score for Ulcerative Colitis; SHS, Short Health Scale; UCCIS, Ulcerative Colitis Colonoscopic Index of Severity; UCEIS, Ulcerative Colitis Endoscopic Index of Severity.

## Discussion

In this study we examined the performance of several endoscopic descriptors of colonic inflammation as well as the most established endoscopic indices for UC; and based on these analyses we propose a new simplified endoscopic score, named the SES-UC.

We believe that both disease extent and the highest degree of inflammatory activity are important pieces of information for an accurate and comprehensive depiction of the inflammatory activity and disease burden in patients with UC. We found that the descriptors *vascular pattern* and *ulcers* perform well in terms of intra- and interobserver agreement, reflect inflammatory activity well, are easy to use, and when used in combination they cover the whole spectrum of disease severity making the addition of a third descriptor redundant. Based on these observations, the two descriptors *vascular pattern* and *ulcers* were used to construct a new simplified endoscopic score, the SES-UC (**Figure 2**), which performed equally well or better than the previously established endoscopic indices examined in this study.

Given that currently available endoscopic indices for UC are not sufficiently accurate, reliable, expedient, and/or easy to use, the aim of this study was to design a new index which embraces all these qualities. We constructed a simple index, herein named the SES-UC, where the scores of only the two descriptors *vascular pattern* (scored 0-2) and *ulcers* (scored 0-3) are added to denote the disease activity of the specific segment evaluated. In conjunction, the results showed that these two descriptors covered the entire range of disease severity and performed well in intra- and interobserver agreement analyses. Importantly, the results showed a strong and statistically significant correlation between the SES-UC and the histological degree of disease activity. Finally, the SES-UC_Σ_ score (the sum of the SES-UC scores from each of the five large bowel segments) displayed a strong and statistically significant correlation with both fecal calprotectin and plasma CRP levels. Since the SES-UC is easier to use than other indices, these results make the SES-UC an appealing endoscopic index to be considered for clinical practice as well as for research studies.

Previously developed endoscopic indices have been designed to generate a single number that denotes either the highest degree of inflammation on a sigmoidoscopy or tries to capture the entire colonic disease burden. The latter includes both disease extension and degree of activity, and often an attempt is made to balance these two dimensions by reporting them as a quotient of some type. The underlying problem remains however since a quotient does not reveal the magnitude of the dividend and divisor but only their relative size relationship. The single number version of the SES-UC (see below for additional details and rationale) is afflicted with a similar problem in certain clinical situations, *i.e.* patients with differential disease patterns (*e.g.*, severe proctitis versus mild pancolitis) may score similarly when the SES-UC_max_ and SES-UC_Σ_ are multiplied. To report the disease status observed by colonoscopy in a clinical context we therefore suggest that two numbers should be used, one that depicts the maximum inflammation found, and one that depicts the total colonic inflammatory burden. A practical way to convey these two numbers would be to write them as SES-UC_max_ followed by SES-UC_Σ_ in parenthesis, where SES-UC_max_ is the maximum SES-UC score found and SES-UC_Σ_ is the sum of the five large bowel segment-specific SES-UC scores. Thus, if a colonoscopy would show a SES-UC score of 5 for the rectum and the sigmoid colon, and a SES-UC score of 3 for the descending, the transverse, and the cecum/ascending colon, the colonoscopy would be reported as SES-UC 5(19). In this way both these important dimensions of the disease are captured and communicated. Indeed, use of the SES-UC_max_ score was supported by the results that showed statistically significant correlations between the SES-UC_max_ and the highest degree of histological inflammation, fecal calprotectin levels, and plasma CRP levels, respectively. If only a sigmoidoscopy or an incomplete colonoscopy is performed, we suggest stating the SES-UC_max_ and that the SES-UC_Σ_ is reported as “X” to avoid misunderstandings and to communicate in an easy way that only a sigmoidoscopy was done. Taking the example from above it would be reported as SES-UC 5(X). For an overview of the SES-UC with calculation examples, see **Figure 2**.

Thus, when using the SES-UC in a clinical context, both numbers (*i.e.*, the SES-UC_max_ score and the SES-UC_Σ_ score) should be presented together, side by side, to communicate the amplitude of disease activity as well as the total inflammatory burden, since both are important pieces of information for the clinical management of a specific patient. However, in clinical trials where the purpose is to evaluate the degree of efficacy of a therapeutic intervention, the endpoint is analyzed and presented at the group level and relates to a change from baseline (before the therapeutic intervention). In placebo-controlled trials or non-inferiority trials, most often the endpoints are assessed using non-paired tests comparing groups, and since subjects are randomized to the various groups, the issue described above will be similarly represented in the groups. In cases where the study-design involves a paired test, evaluating the change in disease activity (including amplitude and extent) from baseline to the time of readout in each subject, the single number SES-UC will change regardless of the disease pattern of the individual subject if the therapeutic intervention is efficacious. In addition, the use of a single number (rather than two numbers presented side by side) will be more manageable in statistical analyses. Thus, for the purpose of clinical trials where the evaluation of therapeutic efficacy by means of statistical analyses is the primary objective as opposed to the more complex communication of several clinically crucial dimensions of the disease, we suggest converting the two numbers to a single number by multiplying the SES-UC_max_ score by the SES-UC_Σ_ score. Using the example from above again, the result would be 5x19 = 95. However, this single number version of the SES-UC is a preliminary score proposal which needs to be investigated and validated in future studies.

The development of the UCEIS involved a methodologically solid approach and it is thus one of the few indices that is properly validated (11, 12). However, the selection and the number of descriptors were not re-evaluated in a validation cohort after the initial study (11, 12). The UCEIS has received broad recognition, but nevertheless the MES is still by far the most frequently used index (2). The development of the UCCIS was, similarly to the UCEIS, based on a meticulous process, but the index has the additional downside that it entails a rather complicated calculation to arrive at the final score which makes it less feasible for use in clinical practice. Nevertheless, the UCCIS performed overall slightly better than the other indices in our study, including the SES-UC. However, the UCCIS includes the descriptor *granularity* since it performed well in the developmental work regarding interobserver agreement and prediction of a global assessment of endoscopic severity. However, in our study it became evident during the consensus meeting that was held for discussing interpretations of descriptor definitions, that the grading of *granularity* is ambiguous. The various types and degrees of *granularity* (i.e., granular mucosa, fine granularity, coarse granularity, smooth granularity, sandpaper-like granularity, and nodularity) are poorly defined and difficult to interpret (11, 13, 37). In addition, it is rather common that the mucosa, despite being evidently inflamed, does not display true elements of endoscopic granularity, which entices to use the granularity descriptor as a measure of the overall disease activity on a three-level scale (normal, some, or marked) which may explain its fairly good agreement statistics in some studies including ours (13). We speculate that these are reasons why 6 out of 9 endoscopic UC-indices (including the MES and the UCEIS) reviewed by Lee et al. in 2019 did not comprise the descriptor *granularity* (38).

The MES, although broadly used both in clinical practice and clinical trials, has never been properly validated. It comprises six descriptors that each are mentioned to be either absent, mildly present, or markedly present at the four levels of disease severity. The various MES-levels are not distinctly defined in terms of which descriptors should be confirmed or negated, or how many descriptors that need to be confirmed for a given mucosal appearance to qualify for one or the other MES-level. Nor does the MES come with guidance on whether some of the descriptors are dominant over the others, or if some are mandatory while others are not, for a certain MES-level. Furthermore, it is our impression that evaluations of the mucosa when using the MES are not truly based on a joint assessment of the various descriptors that are actually included in the index, but rather based on the individual assessor’s general perception of what remission, mild, moderate, and severe disease looks like endoscopically. All of these caveats together likely explain the high degree of interindividual variability observed for this score. Indeed, studies on the MES have shown that agreement on the level of disease severity among observers is present only in 21% of patients (39), that agreement coefficients may be as low as 0.11 (40), and that agreement numbers may be lower among experienced as compared with unexperienced endoscopists (41). Our kappa-data for the MES are considerably higher than in these and other studies reporting on the MES, which we suspect is due to the circumstance that we, prior to the study, had discussed the MES in depth and as part of the study procedure we included a consensus meeting among the assessors to align how to use and to score the MES. As a final comment, the MES depicts only the most inflamed part of colon on a sigmoideoscopy and does not take the extent of the disease into account, similarly to the UCEIS.

There are indeed numerous endoscopic indices available for the evaluation of UC, and they all have the common feature that they are constructed from a number of descriptors that depict various signs of colonic inflammation (42). In the developmental work underlying the UCCIS and the UCEIS, the performance of 12 and 11 descriptors, respectively, was examined (altogether 17 different descriptors) (11, 13). Based on the performance-data, the research-groups selected four and three descriptors, respectively, (altogether 5 different descriptors) to construct a new index. Instead of re-evaluating all 17 descriptors, we chose to focus on the 5 descriptors that had been deemed the best in these previous studies and added *erythema* which is included in the MES (1). In our study, all assessed descriptors (i.e. *erythema*, *vascular pattern*, *granularity*, *friability*, *bleeding*, and *ulcers*) performed overall well and similarly in terms of intra- and interobserver agreement. Thus, the decision of which descriptors to combine into a composite endoscopic score should not be based solely on these factors. From experience, different descriptors seem to be more or less sensitive in mild, moderate, and severe disease, respectively, and thus provide good discriminatory abilities at various parts of the inflammatory spectrum. This was also suggested by some of the data produced during the development of the UCEIS (11). To examine whether a given descriptor is a good discriminator within the realm of mild, moderate, and severe inflammation, respectively, we examined how the respective descriptors were scored at various degrees of disease activity as defined by the MES. Our results showed that *vascular pattern* could differentiate various levels of low-grade inflammation whereas *ulcers* was the best discriminator in moderate and at high-grade inflammation.

In the current study, all investigated indices performed overall well regarding intra- and interobserver agreement, with the UCCIS displaying slightly higher *κ* values than the other established indices. Furthermore, the UCCIS was the only index among the established indices that correlated to fecal calprotectin which merits further investigation since there is an increasing use of fecal calprotectin as a triaging tool to determine the need for an endoscopic examination. All established indices showed a strong correlation to levels of plasma CRP and histological disease activity, respectively. The latter is important considering the trend toward an even more strict definition of mucosal healing that includes histologic remission, sometimes referred to as complete remission (43). On the contrary, all endoscopic indices correlated poorly to symptom levels (SCCAI) which was expected since these include subjective parameters that do not necessarily mirror the degree of inflammatory activity (44). Previous studies have also shown that symptom levels correlate poorly with the endoscopic appearance, and that symptoms may persist despite lack of endoscopic inflammation as well as the opposite (45, 46). These observations differ from those of the group that developed the UCCIS, where endoscopy was found to correlate well with symptomatic disease activity (14). In recent years, patient reported outcome measurements in UC have focused on stool frequency and blood in stools (PRO-2) which are considered to more accurately reflect the level of colonic inflammation, and it is thus possible that PRO-2 correlates better with endoscopic indices than SCCAI (47).

Mucosal inflammation in UC is not always continuous on endoscopic examination and in addition, the highest degree of inflammatory activity may be found proximally to what can be visualized by means of a sigmoidoscopy. These features are not that uncommon and may be seen in atypical disease phenotypes or as a secondary phenomenon to medical therapy. Furthermore, for the purpose of detecting potential dysplasia or neoplasia, sigmoidoscopy is of limited use. Therefore, in many situations, assessment of the entire colon may be preferable as compared to sigmoidoscopy. We calculated the proportion of patients for which the most inflamed part of the colon was situated proximally to the sigmoid colon and found that this occurred in a non-negligible proportion of cases ranging from 25-38% depending on which endoscopic index was used. These findings are corroborated by previous studies including that by Kato et al. which found that 14% of 545 UC-patients had their maximum inflammation on the oral side of the splenic flexure, as well as other studies demonstrating patchiness in UC (16, 48, 49). The topic of whether a complete colonoscopy is preferable over sigmoidoscopy is a matter of debate (19, 50). Our data suggest that the most severely affected segment is located in the proximal half of the colon in a considerable proportion of patients, which in turn indicates that there is a risk of underestimating the disease severity if only a sigmoidoscopy is performed. Also, for the purpose of clinical trials and other scientific studies, in order to correctly quantify the patient’s disease burden, a full colonoscopy may be preferable (9, 51). Still, sigmoidoscopy may be an adequate choice of endoscopic examination in certain cases, i.e. patients with limited distal disease where symptoms or biomarkers do not suggest progression in disease extent; patients with acute severe colitis where the risk for endoscopic complications may be increased; and patients where dysplasia or neoplasia are not suspected or sought for. Undoubtedly, compared to a full colonoscopy, a sigmoidoscopy is performed more swiftly, is less burdensome for both the patient and the healthcare system, and will therefore – despite its caveats – continue to be a cornerstone examination in UC.

There are some endoscopic indices for UC disease activity including the Modified Mayo Endoscopic Score that account for the proportion within a colonic segment being inflamed. However, this approach infers an increased complexity and the exact disease extent in terms of centimeters is probably not a crucial aspect to consider in disease evaluation, particularly not in a clinical context. This notion is corroborated by the extent patterns that are considered to be clinically relevant to differentiate according to the Montreal classification, *i.e.*, proctitis, left-sided colitis, and extensive colitis. In Crohn’s disease on the other hand, which displays highly differential extent patterns compared to UC, it may be more relevant and important to take the extent of the inflammatory changes within each bowel segment into account, which is indeed reflected by the CDEIS and the SES-CD. Also, the amplitude of inflammatory activity in UC is more prone to earlier change than the extent after a new treatment has been initiated. We believe that it may be relevant to quantify the within-segment extent more exactly in disease monitoring over time to evaluate progress of disease extent and potentially when determining which patients that should be subject to endoscopic surveillance. However, when designing an endoscopic index, trying to strike the optimal balance between simplicity/utility and accuracy/reliability we believe that the within-segment extent may represent a too high level of detail to be included.

When comparing the SES-UC with the currently available indices we conclude that the SES-UC does not necessarily perform better than the previously established indices, but rather similarly. When taking simplicity into account, which is crucial in clinical practice, we would like to argue that the SES-UC is a strong candidate index displaying non-inferiority in performance in combination with enhanced simplicity in comparison with the previously established indices.

The UCEIS comprises the descriptor bleeding in addition to vascularity and ulcers. Bleeding has a similar discriminatory profile as ulcers and the two correlate very closely (*r*_s_ 0.86; p<0.0001), making it likely that *bleeding* does not contribute with substantial additional information over and beyond *ulcers*. Nevertheless, in the UCEIS developmental study the addition of *bleeding* to *vascularity* and *ulcers* gave a better correlation with the subjective visual analogue scale judgment of overall disease severity which prompted the authors to include *bleeding* in the final UCEIS index (11). However, the actual selection of descriptors and the specific number of descriptors included in the UCEIS have not been addressed in a separate validation study (11, 12). The descriptor *bleeding* should be scored at intubation prior to instrument-contact with the mucosa, but since endoscopic evaluation routinely is done during withdrawal it is conceivable that confusion and misunderstandings may arise (52). Indeed, this very problem was brought up in the UCEIS validation study as a potential explanation as to why *bleeding* displayed lower *κ* values than *vascularity* and *ulcers*, and furthermore the scoring of *bleeding* changed more than for the other descriptors when the investigators were informed about the patient’s symptoms (12). Also, *bleeding* is part of the evaluation of the descriptor *friability* (as is the case for the UCCIS), which may be further divided into *incidental friability* and *contact friability*, and in the developmental process for the UCEIS, *friability* was excluded due to high levels of variability (11). *Bleeding-friability* is included in the UCCIS but due to its performance it has the lowest weighting factor among the four descriptors included (13). Taken together, it may be argued that *bleeding* is an endoscopic descriptor with caveats that may be appropriate to exclude from an endoscopic index.

It may be challenging to identify a suitable independent gold standard measurement of disease activity against which the performance of the individual endoscopic descriptors ought to be compared. We note that Travis et al. and Thia et al. encountered this difficulty when developing the UCEIS and the UCCIS, respectively, and that both groups used a visual analogue scale as reference (11, 13, 14). A visual analogue scale may be criticized for being a highly subjective reference instrument, the avoidance of which paradoxically has been one of the main reasons for developing these new more objective endoscopic scoring systems. In addition, the visual analogue scale assessment is not independent of the descriptors, but rather the opposite since it is largely based on the descriptors being evaluated. Instead, histology may perhaps be the best reference to use and should probably be considered as the gold standard in this context. However, histological analyses are not caveat-free; these too are based on subjective assessments (which could potentially be mitigated by use of machine learning based histological evaluation) and furthermore may not always reflect the macroscopic appearance since biopsies are collected in specific locations which may not be representative. This said, we consider the use of histopathological analysis as reference in accuracy tests a strength of this study, as compared to using a visual analogue scale.

The study has several limitations. Firstly, it included a limited number of patients and assessors. Although our study entailed the reading of one hundred unique colonic segments by four different readers on three different occasions scoring six descriptors, generating a total of 8400 unique data-points, the study is still rather small, and larger studies are needed to draw firm conclusions. Secondly, it was performed at a single center. Lastly, the MES was used as reference to test how well parameters discriminate between mild, moderate, and severe inflammation.

In summary, we investigated individual descriptors of colonic inflammation in UC and found that *vascular pattern* and *ulcers* were good as well as sufficient at discriminating inflammatory degrees at both low and high inflammatory disease activity levels, respectively. The data from this study suggest that the MES, the UCEIS, and the UCCIS perform similarly as instruments for evaluating the level of disease activity in UC, with the UCCIS perhaps being slightly better in some parameters as well as accounting for disease extent which the MES and the UCEIS do not. We propose a new simplified endoscopic index, the SES-UC, which is based on the two descriptors *vascular pattern* and *ulcers*. The results demonstrated that the SES-UC performed equally well as the other indices, and in some respects even better, which makes it a good candidate index for use in both clinical practice and clinical trials. For clinical purposes we suggest the SES-UC index to be reported using two numbers; one for the maximal segmental score (SES-UC_max_) and one for the sum of all five large bowel segmental scores (SES-UC_Σ_) in parenthesis as follows: SES-UC_max_(SES-UC_Σ_). For clinical trial purposes we provisionally propose that the two numbers are multiplied to generate a single number; a formula which performed very well in terms of reflecting the degree of objectively quantified disease activity and which is more expedient for statistical processing. Additionally, our results suggest that sigmoidoscopy is insufficient in a non-negligible number of patients. Finally, larger studies are needed to evaluate and validate the SES-UC index and to define cut-off levels for various degrees of disease severity.

## Data Availability

The raw data supporting the conclusions of this article will be made available by the authors, without undue reservation.
